# Correlates of physical activity among people living with and without HIV in rural Uganda

**DOI:** 10.3389/frph.2023.1093298

**Published:** 2023-07-20

**Authors:** Smart Z. Mabweazara, Jennifer Manne-Goehler, Prossy Bibangambah, June-Ho Kim, Sentongo Ruth, Linda C. Hemphill, Samson Okello, Mark Hamer, Mark J. Siedner

**Affiliations:** ^1^ Clinical Research Department, Africa Health Research Institute, KwaZulu-Natal, South Africa; ^2^ Department of Medicine, Harvard Medical School, Boston MA, United States; ^3^ Department of Medicine Brigham and Women's Hospital Boston, MA, United States; ^4^ Faculties of Medicine and Radiology, Mbarara University of Science and Technology, Mbarara, Uganda; ^5^ Ariadne Labs, Brigham and Women's Hospital, Boston, MA, United States; ^6^ Department of Medicine, Massachusetts General Hospital, Boston, MA, United States; ^7^ Department of Epidemiology, University of North Carolina, Chapel Hill, NC, United States; ^8^ Division of Surgery & Interventional Science, University College London, London, United Kingdom; ^9^ Faculty of Medical Sciences, University of KwaZulu-Natal, Durban, South Africa

**Keywords:** physical activity, HIV, correlates, cardiovascular disease, socioeconomic status

## Abstract

**Background:**

Antiretroviral therapy (ART) has led to diminishing AIDS-related mortality but a concomitant increase in non-communicable diseases (NCDs) for people with HIV (PWH). Whereas physical activity (PA) has been shown to help prevent NCDs and NCD outcomes in other settings, there are few data on PA and its correlates among PWH in high-endemic settings. We aimed to compare PA by HIV serostatus in rural Uganda.

**Methods:**

We analysed data from the UGANDAC study, an observational cohort including PWH in ambulatory HIV care in Mbarara, Uganda, and age- and gender-matched people without HIV (PWOH). Our primary outcome of interest was PA, which we assessed using the International Physical Activity Questionnaire and considered as a continuous measure of metabolic equivalents in minutes/week (MET-min/week). Our primary exposure of interest was HIV serostatus. We fit univariable and multivariable linear regression models to estimate the relationship between HIV and PA levels, with and without addition of sociodemographic and clinical correlates of PA (MET-min/week). In secondary analyses, we explored relationships restricted to rural residents, and interactions between gender and serostatus.

**Results:**

We enrolled 309 participants, evenly divided by serostatus and gender. The mean age of PWH was 52 [standard deviation (SD) 7.2] and 52.6 (SD 7.3) for PWOH. In general, participants engaged in high levels of PA regardless of serostatus, with 81.2% (251/309) meeting criteria for high PA. However, PWOH reported higher mean levels of PA met-minutes/week than PWH (9,128 vs 7,152, *p *≤ 0.001), and a greater proportion of PWOH (88.3%; 136/154) met the criteria for high PA compared to PWH (74.2%; 115/155). In adjusted models, lower levels of PA persisted among PWH (*β* = −1,734, 95% CI: −2,645, −824, *p* ≤ 0.001). Results were similar in a sensitivity analysis limited to people living in rural areas.

**Conclusion:**

In a rural Ugandan cohort, PWOH had higher levels of PA than PWH. Interventions that encourage PA among PWH may have a role in improving NCD risk profiles among PWH in the region.

## Introduction

Antiretroviral therapy (ART) has led to a narrowing gap in life expectancy between people with HIV and people without HIV (1, 2). As AIDS-related mortality has decreased, people with HIV are at increasing risk of non-communicable diseases (NCDs), including cardiovascular disease (CVD) (3–5). In the global north, people with HIV have been observed to have a 1.5- to 2-fold greater risk of CVD than people without HIV (5). A combination of determinants is thought to cause this increased risk, including traditional CVD risk factors (6), HIV-specific factors such as chronic immune activation and inflammation (7, 8), ART-related dyslipidemia and other metabolic comorbidities (9, 10), behavioural factors such as smoking and physical inactivity (11–13) and inequalities in access to health care (14–16). A particular concern is the effect of modern ART regimens, such as dolutegravir- to cause significant increases in weight, especially when in combination with tenofovir alafenamide fumarate (17).

The rising tide of CVD morbidity among people with HIV necessitates exploration of scalable interventions that can help improve health, particularly in high-burden HIV endemic settings. There is an abundance of evidence showing the health-related benefits of physical activity (PA) in the general population, including decreasing the risk of CVD among people with HIV (18). Physical activity has multiple benefits, including reducing many modifiable CVD risk factors that are increased because of HIV infection and/or treatment (19, 20). For example, benefits of PA in CVD risk reduction include elevated serum HDL-C, decreased triglycerides and total cholesterol (21, 22), and a reduction in BMI, waist-to-hip ratio, and total body fat (23). People with HIV who regularly engage in PA also have significantly lower advanced glycation end products, lower waist circumference (WC) (24), and less lipodystrophy than non-active people with HIV (25). In people with HIV, PA has been beneficial for maintaining good health (26), improving metabolic profiles and cardiorespiratory fitness (27), maintaining lean muscle tissue, improving cardiopulmonary function and immune status, enhancing quality of life (28), controlling inflammation, and improving cardio-metabolic health (27, 29).

However, despite the numerous known benefits of PA for people with HIV and for CVD risk reduction, and its potential as a focus of health interventions, there is little data on PA levels among people in HIV endemic settings, particularly in sub-Saharan Africa. There is substantial inter- and intra-regional variation in PA, and PA also differs significantly by social determinants (30–33). Furthermore, since HIV serostatus presents unique barriers to PA among rural or underserved people with HIV (34), comparing PA levels and determinants to people without HIV will also be a crucial step towards designing PA interventions for this population.

Thus, our overarching aim was to describe the distribution and determinants of PA in a cohort of adult people with HIV on ART and age-matched people without HIV in Uganda. We additionally aimed to explore how PA differs by demographic and cardiometabolic factors, and whether these factors confound relationships with HIV serostatus. We hypothesize that people with HIV engage in lower levels of PA and lower intensity PA than people without HIV and that, women, individuals who are older, those with higher blood pressure and HbA1c ≥ 7 and of low educational attainment, wealth quartile and living in an urban location will also engage in less PA.

## Materials and methods

### Population and study design

The UGANDAC study was a longitudinal observational cohort study of people with HIV in ambulatory HIV care in Mbarara, Uganda, and a group of age- and gender-matched people without HIV comparators (NCT 02445079) (35). The objective of the study was to test hypotheses related to associations between HIV and NCD risk, including cardiovascular and pulmonary disease, in an HIV-endemic setting in sub-Saharan Africa in an aging population. Thus, our study uses a population of people aged 40 years of age and older. To be included in the study, people with HIV had to meet the following criteria: (1) in ambulatory care at the Immune Suppression Syndrome Clinic at Mbarara Regional Referral Hospital, (2) age ≥40 years and (3) duration of ART use ≥3 years (36, 37). After enrolment of people with HIV, age and gender-matched people without HIV comparators living in the clinic catchment area were recruited from their homes using a population census. Since this was a longitudinal study, for this study we use data from visit one (i.e., first year of the study). The only data which we carried forward were for wealth quartile since this data only started to be collected in visit two (i.e., in the second year of the study). Since this was a secondary data analysis from a parent cohort study. The parent study was initially powered to test for a difference in the progression of carotid intima media thickness by HIV serostatus in Uganda (35). In keeping with best practices, a post-hoc power calculation was not conducted for this analysis, given their known pitfalls and strong recommendations from experts in the field not to be undertaken in this scenario (38).

### Data collection

Trained study nurses conducted interviews between December 2013 and May 2018 to capture sociodemographic data, history of CVD diagnosis and its management, CVD risk factors (including, hypertension, diabetes mellitus, atherosclerosis and dyslipidemia) (39). Study nurses also measured weight using standardized scales (seca 762, Hanover, USA) and height using roll-up measuring stadiometers (seca 206, Hanover, USA) (39). Height was measured to the nearest 0.1 cm and weight was measured to the nearest kilogram (39). Height and weight were then used to calculate body mass (BMI) as weight (in kilograms) divided by the square of height (in meters), and categorized BMI as underweight (30 kg/m^2^), normal weight (18.5–24.9 kg/m^2^), overweight (25–29.9 kg/m^2^), or obese (>30 kg/m^2^) (39). Bilateral, resting blood pressure was collected using automated digital upper arm sphygmomanometers (Omron Healthcare Inc., Bannockburn, USA) with small (<21 cm), normal (22 cm–32 cm), and large cuffs (35 cm–44 cm). This was done with participants seated in a chair and allowed to rest for 5 min. The average of the measurements from both arms was used to determine the blood pressure of each participant (39). Blood for HBA1c testing was collected using Siemens Vantage A1c testing kits (Siemens Medical Solutions USA, Malvern, PA) (40). To measure relative wealth in our study population, we collected data on ownership of assets as described previously and used in Demographic Health Surveys across the region (41). In summary, we used principal component methods and selected the first principal component applied to multiple binary indicators for household-owned assets (e.g., bicycle, radio) and housing characteristics (e.g., type of toilet facilities, water source). We then constructed four quartiles from this variable to enable relative and interpretable comparisons in wealth (lowest, low, high, highest) within the cohort.

Physical activity, the primary outcome of this study was assessed using an adapted version of the International Physical Activity Questionnaire (IPAQ) short form, which was developed to assess PA across all domains of leisure-time, work, transportation, and household tasks (42). It has been previously validated compared to PA (43). Specifically, a study that collected reliability and validity data on the IPAQ short form showed that its reliability is within acceptable levels with 75% of correlation coefficients observed above 0.65 (44). Similarly, using a total of 781 adults, fair to moderate agreement between the IPAQ short form data against accelerometers was shown in the same study (pooled correlation coefficient 0.3 (95% CI: 0.23–0.36) (44). Respondents report frequency and duration of walking, moderate-intensity and vigorous-intensity activity performed for at least 10 min duration per session. Our adapted questionnaire captured PA relating to activities of daily living such as work and travel-related PA which were specific to the Ugandan rural population context. As such, activities which were captured were a combination of aerobic and anaerobic activity of low and moderate-to-vigorous intensity.

### Statistical analysis

We analysed the first visit for each participant with available data on PA. Descriptive statistics were used to summarize sociodemographic (age, sex, educational attainment, wealth quartile, and residential location) and clinical factors (blood pressure, HbA1c, and BMI) by HIV serostatus. These were reported as mean [standard deviation (SD)] or median and interquartile range (IQR) for continuous variables and as frequencies (n and percent) for categorical variables. Measures of association by HIV serostatus were computed using non-parametric log-rank testing for continuous variables and Pearson chi squared testing for categorical variables.

Our outcome of interest was PA. To estimate PA levels, we first calculated the weekly minutes of walking, moderate-intensity and vigorous-intensity activity by multiplying the number of days/weeks by the duration on an average day. Reported minutes per week in each category were weighted by a metabolic equivalent (MET; multiples of resting energy expenditure) resulting in a PA estimate independent of body weight, expressed in MET-minutes/week and calculated by multiplying METs by minutes/week (44). Physical activity was defined in two ways: (1) for our primary analysis we considered PA as a continuous variable (MET-minutes/week) and (2) for a pre-specified secondary analysis, we dichotomized PA into high PA versus low or moderate PA. To do so, we used standardized criteria (42), in which we considered walking to be 3.3 METS, moderate PA to be 4 METS and vigorous PA to be 8 METS and 2) categorized as “low” (physically inactive), “moderate” and “high” physical activity, based on the following definitions: Low: Meets neither ‘moderate’ nor ‘high’ criteria; Moderate: Meets any of the following three criteria: (a) 3 days of vigorous activity of at least 20 min/day; (b) 5 days of moderate-intensity activity or walking of >30 min/day for >10 min at a time; or (c) 5 days of any combination of walking, moderate-intensity or vigorous-intensity activities achieving at least 600 MET-minutes/week; High: Meets either of two criteria: (a) vigorous-intensity activity on >3 days/week and accumulating at least 1,500 MET-minutes/week; or (b) >5 days of any combination of walking, moderate-intensity, or vigorous-intensity activities achieving at least 3,000 MET-minutes/week. We adapted the questionnaire to reflect the rural Ugandan context. In the adapted PA questionnaire, vigorous activities included cutting or carrying wood, cutting crops, shovelling, grinding or pounding millet, or digging. Moderate physical activities included carrying water or a load on your head while walking, scrubbing, washing clothes, or tending animals. These activities were categorised according to the compendium of physical activities.

For our primary analysis we considered PA as a continuous measure of MET-minutes/week. We fitted univariable and multivariable linear regression models, with HIV as the primary exposure of interest, with and without additional confounding variables, including sociodemographic (age, gender, educational attainment, residential location, and wealth quartile) and clinical factors (HbA1c, blood pressure, and BMI). We used purposeful selection (45) in the regression model and included all items in the univariable model with a *p*-value of <0.25 in the multivariable model and included HIV in both models *a priori* because it was our primary explanatory variable of interest. In pre-specified secondary analyses, we then repeated these steps, but with analysis restricted to individuals living in rural areas because most people without HIV participants in the cohort were recruited from a rural location**.** We also repeated these steps to identify those with the highest level of PA, by dichotomizing PA as either high or low as the outcome of interest and fitting logistic regression models to assess associations with HIV and the same confounding variables as above. Further, analysis was also done to identify correlates of PA with interaction terms on gender and serostatus. All analyses were conducted using Stata version 16 (College Station, TX).

### Ethical considerations

Study procedures were reviewed and approved by the Institutional Review Board of Mass General Brigham (2014P001928) and the Research Ethics Committee of the Mbarara University of Science and Technology (06/04–14). All participants gave written informed consent. Permission to conduct this secondary data analysis was also granted by the University of KwaZulu Natal Biomedical Research Ethics Committee (BREC/00003396/2021).

## Results

### Study population

Sociodemographic, PA and clinical factors of participants are summarized in Table 1. Of the 309 participants in our study, approximately half (155/309, 50%) of the participants were people with HIV and the cohort was nearly evenly divided by sex [158/309 (51%) male]. Most participants had primary or less education (255/309, 83%) and most lived in a rural residential location (263/309; 85%). People with HIV tended to have higher asset ownership, with a greater proportion of people in the highest quartile (34% vs 13%) and lower proportion of people in the lowest quartile (20% vs 32%) of asset ownership (*p* < 0.001). Most participants had a HbA1c < 7, which was similar for people with HIV and people without HIV. The mean systolic and diastolic blood pressures for people with HIV were 114 [standard deviation (SD) 20] and 71.6 (SD 12) respectively whilst that for people without HIV was 120 (SD 18) and 76 (SD 12), respectively. In terms of BMI, a greater proportion (43/155, 28%) of people with HIV were in the overweight/obese BMI category, compared to 37/154, 24% in people without HIV, even though the proportions were statistically similar. In terms of PA, both people with HIV (115/155, 74%) and people without HIV (136/154, 88%), had a greater proportion of people reporting high PA than low or moderate PA and this proportion was higher in people without HIV. For people with HIV, the median CD4 was 458 cell/ml (IQR: 336–559), whilst 86% had a suppressed viral load.

**Table 1:** Cohort sociodemographic and clinical characteristics.

Characteristics	People living with HIV (*n* = 155)	HIV un-infected (*n* = 154)	*p-*value^a^
Age (y) mean (SD)	52.0 (7.2)	52.6 (7.3)	0.443
Missing	0	0	
Sex, *n* (%)	* *	* *	0.70
Men	81 (52.3)	77 (50.0)	* *
Women	74 (47.7)	77 (50.0)	* *
Missing	0	0	* *
Educational attainment, *n* (%)^b^	* *	* *	0.005
Primary school or less	130 (83.9)	125 (81.2)	* *
Secondary school and greater	24 (15.5)	17 (11.0)	* *
Missing	1 (0.7)	12 (7.8)	* *
Location	* *	* *	<0.001
Urban	36 (24.8)	0	* *
Rural	109 (75.2)	154 (100.0)	* *
Missing	10 (6.45%)	0	* *
Wealth quartile, *n* (%)^c^		* *	<0.001
Fewest Assets	31 (20.1)	45 (31.7)	* *
Few Assets	29 (18.8)	46 (32.4)	* *
Middle Assets	42 (27.3)	32 (22.5)	* *
Most Assets	52 (33.8)	19 (13.4)	* *
Missing	1 (0.7)	12 (7.8)	* *
HbA1c, *n* (%)^d^	* *	* *	0.229
<7	146 (94.2)	149 (96.8)	* *
≥7	9 (5.8)	4 (2.6)	* *
Missing	0	1 (0.7)	* *
Blood pressure (mmHg), mean (SD)	* *	* *	* *
Systolic BP*	114.4 (19.5)	119.6 (17.9)	0.0005
Diastolic BP*	71.6 (11.8)	76.4 (11.6)	<0.001
Body mass index category, *n* (%)	* *	* *	0.056
Underweight (<18.5 kg/m^2^)	13 (8.4)	27 (17.5)	* *
Normal (18.5–25 kg/m^2^)	99 (63.9)	90 (58.4)	* *
Overweight/obese (≥25 kg/m^2^)	43 (27.7)	37 (24.0)	* *
Missing	0	0	* *
Physical activity, *n* (%)			0.003
Low PA	13 (8.4)	3 (2.0)	* *
Moderate PA	27 (17.4)	15 (9.7)	* *
High PA	115 (74.2)	136 (88.3)	* *
Missing	0	0	* *
Current CD4 (cell/ml), median (IQR)	458 (336–559)	* *	* *
Viral load detectability, *n* (%)		* *	* *
Detectable	16 (10.32)	* *	* *
Undetectable	133 (85.81)	* *	* *
Missing	6 (10.32)	* *	* *
ART regimen, *n* (%)	* *	* *	* *
AZT/3TC/NVP	93 (60.1)	* *	* *
AZT/3TC/EFV	31 (20.0)	* *	* *
TDF/3TC/EFV	11 (7.1)	* *	* *
Other	20 (12.9)	* *	* *
Missing	0	* *	* *

BP,  blood pressure.

^a^ *p*-values were calculated using χ^2^ for categorical variables and rank sum testing for continuous variables.

^b^ 13 participants (12 HIV-uninfected and 1 HIV-infected) were missing education assessment.

^c^ We used the mean asset index for each person over the course of the study period and 13 participants were missing wealth quartile data.

^d^ One participant had missing data on HbA1c.

* Average of second and third same sitting left and right arm blood pressure measurements; SD, standard deviation; Low PA ≤ 3.3 METS: meets neither “moderate” nor “high” criteria; Moderate PA = Moderate: meets any of the following three criteria: (a) 3 days of vigorous activity of at least 20 min/day; (b) 5 days of moderate-intensity activity or walking of >30 min/day for >10 min at a time; or (c) 5 days of any combination of walking, moderate-intensity or vigorous-intensity activities achieving at least 600 MET-minutes/week; High PA = meets either of two criteria: (a) vigorous-intensity activity on >3 days/week and accumulating at least 1,500 MET-minutes/week; or (b) >5 days of any combination of walking, moderate-intensity, or vigorous-intensity activities achieving at least 3,000 MET-minutes/week; AZT, zidovudine; TDF, tenofovir 3TC = Lamivudine; EFV, efavirenz. N.B: There was no missing data for physical activity.

### Distributions of physical activity by serostatus

**Figure 1:**
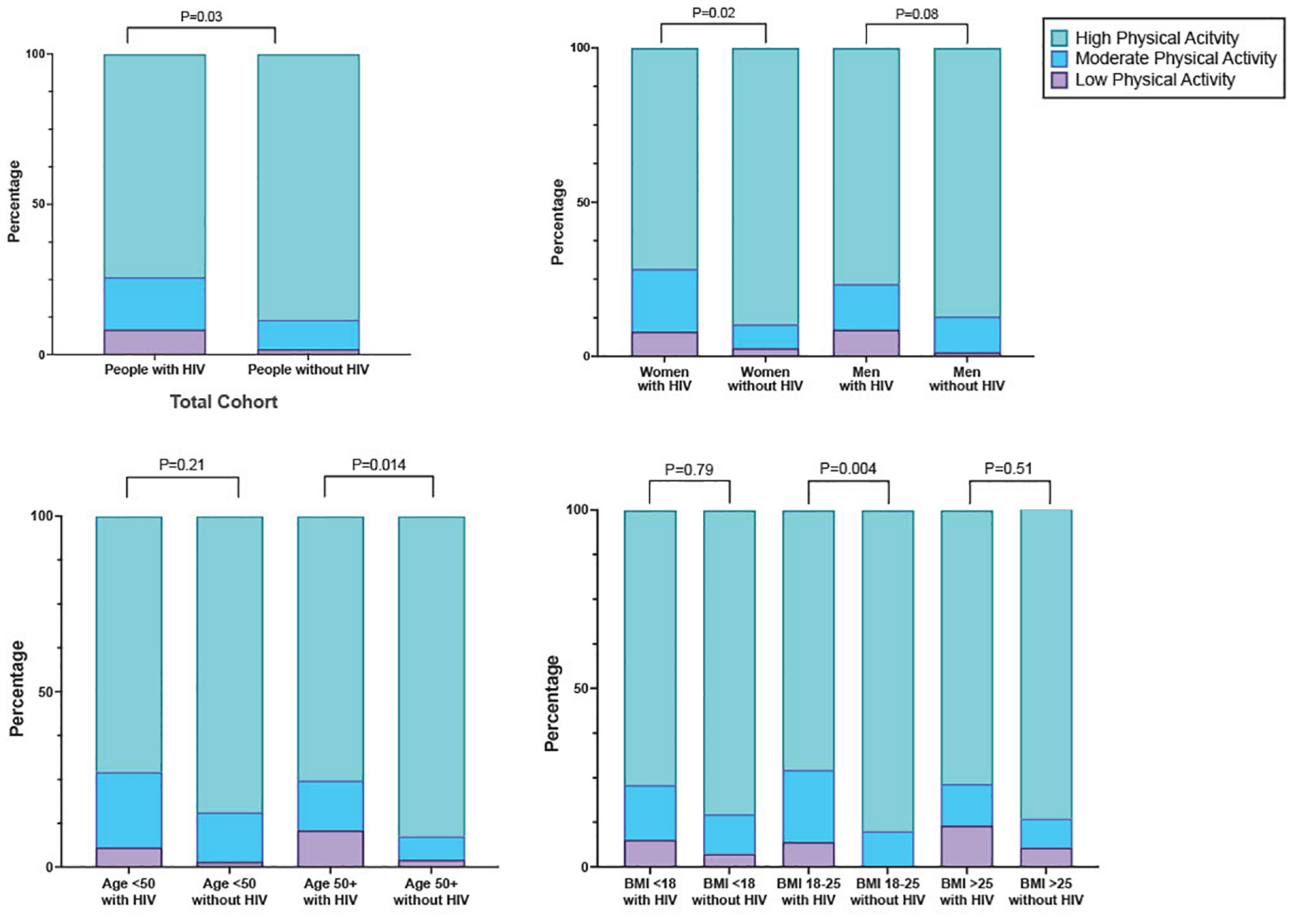
Categorical physical activity level stratified by serostatus, gender, age, and BMI.

**Figure 2:**
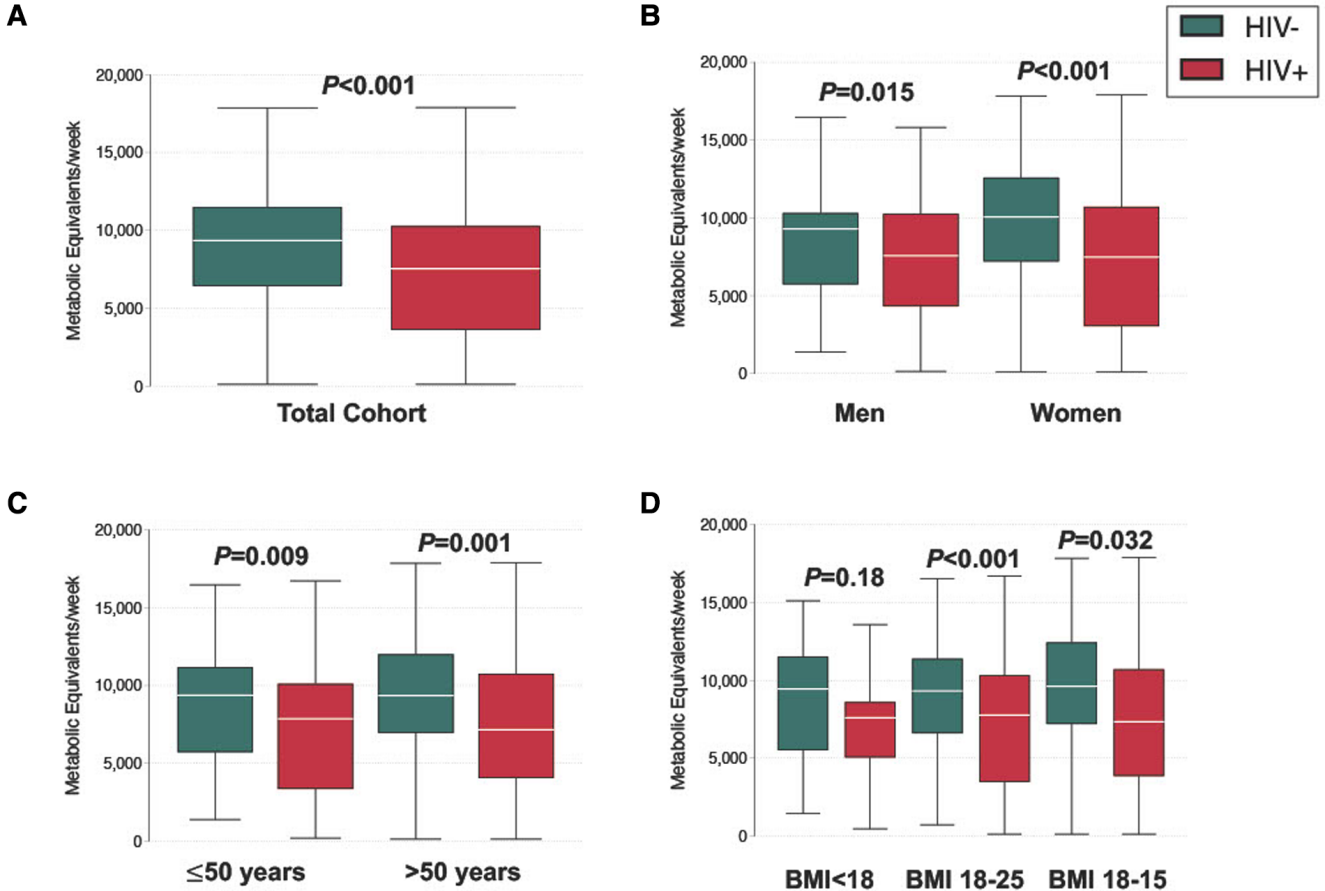
Distribution of physical activity level, stratified by (**A**) serostatus (**B**) gender (**C**) age (**D**) BMI.

In general, the study cohort engaged in high levels of PA, with 81.2% (251/309) meeting criteria for high PA (Figure 1). When we compared PA by HIV serostatus, we found that the mean number of metabolic equivalents/weeks was higher in people without HIV than people with HIV (9,128 vs 7,152, *p* ≤ 0.001), and that a greater proportion of people without HIV (88.3%; 136/154) met the criteria for high PA category compared to people with HIV (74.2%; 115/155, Figure 2A and Table 1). The higher level of PA seen in people without HIV was present in both males and females, across strata of age, and across strata of BMI (Figures 2B–D).

In unadjusted models, having secondary and greater education (*β* = −1,967, 95% CI: −3,340, −595, *p* = 0.005), living in an urban location (*β* = −2,223, 95% CI: −3,632, −814, *p* = 0.002), being in the middle (*β* = −1,531, 95% CI: −2,748, −313, *p* = 0.014) or highest quartile of asset ownership (*β* = −3,934, 95% CI: −5,179, −2,690, *p* < 0.001), having a higher mean diastolic blood pressure (*β* = −450, 95% CI: −841, −68, *p* = 0.021), and being HIV positive (*β* = −19,76, 95% CI: −2,876, −1,076, *p* < 0.001) were significantly associated with lower PA levels (Table 2). In multivariable models, female sex remained significantly associated with higher PA levels (*β* = 931, 95% CI: 49, 1,813, *p* = 0.039), as did being in the highest quartile of asset ownership (*β* = −3,054, 95% CI: −44,348, −1,760, *p* < 0.001), having a higher mean diastolic blood pressure (*β* = −416, 95% CI: −790, −42, *p* = 0.030) and being HIV-positive (*β* = −1,734, 95% CI: −2,645, −824, *p* ≤ 0.001) (Table 2). These results were similar in a sensitivity analysis limited to people living in rural areas (Supplementary Table S1).

**Table 2:** Univariable and multivariable linear regression models to identify sociodemographic and clinical correlates of physical activity in people with and without HIV.

	Univariable Models			Multivariable Model		
	β	95% CI	*p*-value	β	95% CI	*p*-value
Age (each year)	−56	−119, 8	0.087	−24	−85, 36	0.432
Sex						
Male	REF			REF		
Female	612	−313, 1,537	0.194	931	49, 1,813	0.039
Education^a^						
Primary or less	REF			REF		
Secondary and greater	−1,967	−3,340, −595	0.005	−897	−2,211, 418	0.181
Wealth quartile^c^						
Fewest assets	REF			REF		
Few assets	−377	−1,595, 840	0.542	−374	−1,562, 813	0.535
Middle assets	−1,531	−2,748, −313	0.014	−969	−2,177,239	0.116
Most assets	−3,934	−5,179, −2,690	<0.001	−3,054	−4,348, −1,760	<0.001
HbA1c^b^ (each 1%)	21	−453, 496	0.929			
SBP* (each 10 mmHg)	−10	−35, 14	0.4054			
DBP* (each 10 mmHg)	−450	−841, −68	0.021	−416	−790, −42	0.030
Body mass index						
BMI 18.25–25	REF					
BMI < 18.5	−75	−1,489, 1,339	0.917			
BMI ≥ 25 kg/m^2^	−97	−1,672, 1,477	0.903			
HIV serostatus						
PWOH	REF			REF		
PWH	−1,976	−2,876, −1,076	<0.001	−1,734	−2,645, −824	<0.001

SBP, systolic blood pressure; DBP, diastolic blood pressure

^a^ A total of 13 (12 HIV-uninfected and 1 HIV-infected) were missing education assessment.

^b^ One participant had missing HbA1c assessment.

^c^ We used the mean asset index for each person over the course of the study period and a total of 13 participants were missing wealth quartile data.

* Average of second and third same sitting left and right arm blood pressure measurements.

When we considered PA as a categorical variable, with high PA as the outcome, we found that having secondary or greater education (OR = 0.40, 95% CI: 0.19, 0.84, *p* = 0.015), living in an urban location (OR = 0.42, 95% CI: 0.19, 0.92, *p* = 0.030), having the highest quartile of asset ownership (OR = 0.11, 95% CI: 0.04, 0.29, *p* < 0.001), and being HIV-positive (OR = 0.38, 95% CI: 0.21, 0.70, *p* = 0.002) were each 50–90 percent lower odds of high physical activity (Table 3). In adjusted models, being in the highest quartile of asset ownership (OR = 0.12, 95% CI: 0.04, 0.34, *p* < 0.001) remained associated with an approximately 90% lower odds of having high physical activity (Table 3). We did not find evidence of interaction by gender, age, or BMI in models that included interaction terms for each of these (Supplementary Table S2).

**Table 3:** Univariable and multivariable logistic regression models to identify correlates of high physical activity in rural Uganda.

	Univariable Model			Multivariable Model		
	OR	95% CI aOR	*p*	OR	95% CI aOR	*p*
**Age (each year)**	0.98	0.94, 1.02	0.301			
Sex						
Male	REF					
Female	0.95	0.53, 1.67	0.848			
Educational attainment^a^						
Primary or less	REF			REF		
Secondary and greater	0.40	0.19,0.84	0.015	0.64	0.27, 1.53	0.316
Residential location						
Rural	REF					
Urban	0.42	0.19,0.92	0.030	1.15	0.44, 3.01	0.776
Wealth quartile^b^						
Fewest assets	REF			REF		
Few assets	0.83	0.27, 2.60	0.753	0.80	0.23, 2.76	0.723
Middle assets	0.62	0.21,1.83	0.387	0.61	0.19, 1.95	0.404
Most assets	0.11	0.04, 0.29	<0.001	0.12	0.04, 0.34	<0.001
HbA1c^c^ (each 1%)	1.05	0.76, 1.43	0.772			
Systolic BP* (each 10 mmHg)	1.00	0.90, 1.19	0.824			
Diastolic BP* (each 10 mmHg)	0.88	0.69, 1.11	0.278			
Body Mass Index category, *n* (%)						
18.5–25 kg/m^2^	REF					
<18.5 kg/m^2^	1.11	0.45, 2.71	0.820			
≥25 kg/m^2^	1.02	0.52,1.20	0.955			
HIV serostatus						
PWOH	REF			REF		
PWH	0.38	0.21, 0.70	0.002	0.50	0.23, 1.10	0.084

BP, blood pressure.

^a^ A total of 13 (12 HIV-uninfected and 1 HIV-infected) were missing education assessment.

^b^ We used the mean asset index for each person over the course of the study period and a total of 13 participants were missing wealth quartile data.

^c^ One participant had missing HbA1c assessment.

* Average of second and third same sitting left and right arm blood pressure measurements.

## Discussion

In an observational cohort study from Uganda, including people with HIV on ART and age and gender-matched people without HIV from the same community, we found that people without HIV reported higher mean levels of PA energy expenditure compared to people with HIV and that a greater proportion of people without HIV met criteria for high levels of PA than people with HIV. These results remained consistent after adjusting for gender, age, BMI, and cardiovascular disease risk factors, as well as in a sub-sample limited to residents of rural areas. This finding holds relevance in the context of growing reports of CVD as a primary cause of morbidity and mortality among people with HIV globally (46). Although our data were collected prior to the integrase-inhibitor era, they remain relevant in light of an increasing obesity epidemic among people with HIV taking dolutegravir (47). If confirmed in other settings, these findings signal a need for consideration of PA counselling and behaviour change interventions for people with HIV in the region to help realize the multifaceted beneficial impacts of PA on health for this population (27).

Despite the reported benefits of PA for people with HIV in terms of improving metabolic profile, quality of life (27), and functional status (48), studies of people with HIV in sub-Saharan Africa (SSA) have generally demonstrated poor engagement in PA to improve health (49). Indeed, our finding that most people with HIV engage in less PA compared to people without HIV is consistent with the findings of most other studies conducted in SSA (50–54). Although our study did not explore perceptions or behaviours related to PA, we hypothesize that the observed lower levels of PA among people with HIV may be attributed to context-specific barriers to PA among people with HIV. Other studies in the region have suggested that HIV-related symptoms and medications, community safety, depression, cultural beliefs, lack of knowledge about PA and motivation to engage in PA, and fear of negative physical effects of exercise (34, 55) all may be contributors. With sizeable increases in obesity reported among people with HIV taking integrase-inhibitors based therapies (56), future studies should better elucidate the barriers and facilitators of PA among people with HIV in Uganda to assist in design of interventions to promote PA among people with HIV.

There were a number of secondary findings of interest in our study. For example, we found that the study cohort engaged in comparatively high levels of PA regardless of serostatus. This finding is consistent with a national survey of PA data collected as part of a Ugandan national NCD risk factor analysis, which found that 73% of rural participants had high levels of PA, largely achieved through travel and work-related activities (57). Residing in a rural location has also been found to be associated with higher odds of active transportation (58) and intense agricultural activities and manual work as part of employment (59). We similarly found lower levels of PA among urban-dwelling residents compared to rural-dwelling residents in unadjusted models, although the effect was dampened after adjustment for confounders. A number of studies conducted elsewhere in sub-Saharan Africa have also reported increased PA among rural populations (60–63). However, notably, this pattern is not consistent throughout the continent. For example, in South Africa, individuals in urban settings were more likely to engage in moderate PA than rural participants (64), whilst (65) reported that being from a rural formal location reduced the prospect of engaging in vigorous PA. Similar relationships have also been reported outside the African continent (66), and may support the presence of distinct relationships in urbanicity and PA, by broader socioeconomic status.

We also found that female gender was associated with higher PA levels. We hypothesize that the relationship between female gender and PA is due to our study setting in a largely rural area, and high dependence on the agricultural sector for food (67), in a setting where women contribute the highest share of labour (67). Additionally, in many African countries, rural women often are charged with domestic chores, such as collecting water and firewood, processing and preparing food, travelling, and transporting, and caregiving (68). Notably, our results contrast with numerous studies conducted elsewhere in SSA who report that women engage in less PA compared to men (50, 51, 62–65, 69–72). This may be in part, due to some studies only reporting specific domains of PA such as frequency, intensity, and duration (Craft et al., 2014), and not reporting PA as a whole. For example (64), investigated the prevalence and socio-demographic correlates of PA among adults in urban and rural communities in South Africa, specifically reports that women were less likely to engage in vigorous PA and does not report the low and moderate PA.

Similarly, we observed that people with greater wealth had lower levels of PA. This is in contrast with studies who typically report that individuals of higher socioeconomic status (SES) engage in more PA than those of low SES (e.g.,) (65, 73–76). In general, evidence suggests a positive relationship between an individual's economic resources and PA. However, it has recently been suggested that the observed positive relationship between higher SES and PA is mainly a relationship between leisure-time PA and SES, and may be unique of high-income settings (77). By contrast, in our low-income setting, we suspect the opposite relationship may be driven by inherent PA requirements to participation in an agrarian economy (57).

Finally, we found an association between higher diastolic blood pressure and lower levels of PA. Physical inactivity is a risk factor for development of hypertension (78). Moreover, the beneficial effects of PA on hypertension and reduction in both systolic and diastolic blood pressure are well documented (79–83). Thus, the relationship here is not unexpected, and highlights the need for context-specific PA interventions to help improve CVD risk prevention in this region.

Our study should be interpreted in the context of its limitations. We conducted a cross-sectional analysis, as such causal inference cannot be made. We also used a self-reported measure of PA, which may be prone to social desirability bias and less accurate than some objective measures, such as motion sensors. The IPAQ scale is also meant to be a quantitative measure to estimate the magnitude of PA undertaken, but does not provide contextual information about the types of activities being taken (e.g., aerobic versus anaerobic or PA for pleasure versus occupation). Future work should better describe types of and preferences for PA undertaken by PWH to promote intervention development. Comparisons with other studies are limited due to a paucity of research comparing PA and its correlates between people with HIV and people without HIV in sub-Saharan Africa. Finally, given that about half of our study sample were females of menopausal age, adverse health effects that frequently accompany the menopausal transition, such as increases in body weight and mood changes must be considered when interpreting their PA levels.

In summary, this study highlights differences in PA levels between people with HIV and people without HIV. In particular, the development of effective PA interventions, may entail first understanding the factors that facilitate and hinder PA participation, including those that may be unique to people with HIV in rural locations such as HIV-infection related barriers, those that are a consequence of ART, and socioeconomic factors. Most importantly, the emergence of NCDs among people with HIV also calls for an understanding of NCD risk factors among this population and the use of PA as an adjunct therapeutic measure to minimise these risk factors and prevent NCDs. Future studies are recommended to focus on both PA intensity and PA domain so that researchers are able to develop domain-specific interventions.

## Data Availability

The datasets presented in this article are not readily available because this study was a secondary analysis of data. Requests to access the datasets should be directed to mark.siedner@ahri.org.

## Supplementary material

Supplemental Table 1 Multivariable linear regression models to identify sociodemographic and clinical correlates of physical activity in people with and without HIV restricted to individuals living in rural areas

Supplemental Table 2 Multivariable linear regression models to identify sociodemographic and clinical correlates of physical activity in people with and without HIV living with interaction terms on sex, BMI, age, mean diastolic blood pressure and serostatus

## References

[1] Mills EJ Bakanda C Birungi J Chan K Ford N Cooper CL Life expectancy of persons receiving combination antiretroviral therapy in low-income countries: a cohort analysis from Uganda. Ann Intern Med. (2011) 155(4):209. 10.7326/0003-4819-155-4-201108160-00358 21768555

[2] Bor J Herbst AJ Newell ML Bärnighausen T. Increases in adult life expectancy in rural South Africa: valuing the scale-up of HIV treatment. Science. (2013) 339(6122):961–5. 10.1126/science.1230413 23430655 PMC3860268

[3] Farahani M Mulinder H Farahani A Marlink R. Prevalence and distribution of non-AIDS causes of death among HIV-infected individuals receiving antiretroviral therapy: a systematic review and meta-analysis. Int J STD AIDS. (2017) 28(7):636–50. 10.1177/0956462416632428 26868158

[4] Haacker M Bärnighausen T Atun R. Hiv and the growing health burden from noncommunicable diseases in Botswana: modelling study. J Glob Health. (2019) 9(1):010428. 10.7189/jogh.09.010428 31293781 PMC6607958

[5] Shah ASV Stelzle D Lee KK Beck EJ Alam S Clifford S Global burden of atherosclerotic cardiovascular disease in people living with HIV: systematic review and meta-analysis. Circulation. (2018) 138(11):1100–12. 10.1161/CIRCULATIONAHA.117.033369 29967196 PMC6221183

[6] Clark SJ Gómez-Olivé FX Houle B Thorogood M Klipstein-Grobusch K Angotti N Cardiometabolic disease risk and HIV status in rural South Africa: establishing a baseline. BMC Public Health. (2015) 15(1):135. 10.1186/s12889-015-1467-1 25885455 PMC4335669

[7] Deeks SG. Hiv infection, inflammation, immunosenescence, and aging. Annu Rev Med. (2011) 62(1):141–55. 10.1146/annurev-med-042909-093756 21090961 PMC3759035

[8] Kaplan RC Sinclair E Landay AL Lurain N Sharrett AR Gange SJ T cell activation predicts carotid artery stiffness among HIV-infected women. Atherosclerosis. (2011) 217(1):207–13. 10.1016/j.atherosclerosis.2011.03.011 21492857 PMC3139014

[9] Grinspoon S Carr A. Cardiovascular risk and body-fat abnormalities in HIV-infected adults. N Engl J Med. (2005) 352(1):48–62. 10.1056/NEJMra041811 15635112

[10] Data Collection on Adverse Events of Anti-HIV Drugs (DAD) Study Group. Combination antiretroviral therapy and the risk of myocardial infarction. N Engl J Med. (2003) 349(21):1993–2003. 10.1056/NEJMoa030218 14627784

[11] World Health Organisation. Non communicable diseases. Available at: https://www.who.int/news-room/fact-sheets/detail/noncommunicable-diseases (cited September 16, 2022).

[12] Freiberg MS McGinnis KA Kraemer K Samet JH Conigliaro J Curtis Ellison R The association between alcohol consumption and prevalent cardiovascular diseases among HIV-infected and HIV-uninfected men. JAIDS J Acquir Immune Defic Syndr. (2010) 53(2):247–53. 10.1097/QAI.0b013e3181c6c4b7 20009766 PMC2858978

[13] Freiberg MS Chang CCH Kuller LH Skanderson M Lowy E Kraemer KL HIV infection and the risk of acute myocardial infarction. JAMA Intern Med. (2013) 173(8):614. 10.1001/jamainternmed.2013.3728 23459863 PMC4766798

[14] Freiberg MS Leaf DA Goulet JL Goetz MB Oursler KK Gibert CL The association between the receipt of lipid lowering therapy and HIV status among veterans who met NCEP/ATP III criteria for the receipt of lipid lowering medication. J Gen Intern Med. (2009) 24(3):334–40. 10.1007/s11606-008-0891-7 19127386 PMC2642578

[15] Burkholder GA Tamhane AR Salinas JL Mugavero MJ Raper JL Westfall AO Underutilization of aspirin for primary prevention of cardiovascular disease among HIV-infected patients. Clin Infect Dis. (2012) 55(11):1550–7. 10.1093/cid/cis752 22942209 PMC3491860

[16] Ladapo JA Richards AK DeWitt CM Harawa NT Shoptaw S Cunningham WE Disparities in the quality of cardiovascular care between HIV-infected versus HIV-uninfected adults in the United States: a cross-sectional study. J Am Heart Assoc. (2017) 6(11):e007107. 10.1161/JAHA.117.007107 29138182 PMC5721786

[17] Venter WDF Moorhouse M Sokhela S Fairlie L Mashabane N Masenya M Dolutegravir plus two different prodrugs of tenofovir to treat HIV. N Engl J Med. (2019) 381(9):803–15. 10.1056/NEJMoa1902824 31339677

[18] World Health Organisation. WHO guidelines on physical activity and sedentary behaviour. Available at: https://www.who.int/publications-detail-redirect/9789240015128 (cited September 16, 2022).

[19] Jaggers JR Dudgeon W Blair SN Sui X Burgess S Wilcox S A home-based exercise intervention to increase physical activity among people living with HIV: study design of a randomized clinical trial. BMC Public Health. (2013) 13(1):502. 10.1186/1471-2458-13-502 23706094 PMC3668143

[20] Ozemek C Erlandson KM Jankowski CM. Physical activity and exercise to improve cardiovascular health for adults living with HIV. Prog Cardiovasc Dis. (2020) 63(2):178–83. 10.1016/j.pcad.2020.01.005 32014512

[21] Jones SP Doran DA Leatt PB Maher B Pirmohamed M. Short-term exercise training improves body composition and hyperlipidaemia in HIV-positive individuals with lipodystrophy. AIDS. (2001) 15(15):2049–51. 10.1097/00002030-200110190-00021 11600837

[22] Thöni GJ Fedou C Brun JF Fabre J Renard E Reynes J Reduction of fat accumulation and lipid disorders by individualized light aerobic training in human immunodeficiency virus infected patients with lipodystrophy and/or dyslipidemia. Diabetes Metab. (2002) 28(5):397–404. PMID: 12461477

[23] Roubenoff R Weiss L McDermott A Heflin T Cloutier GJ Wood M A pilot study of exercise training to reduce trunk fat in adults with HIV-associated fat redistribution. AIDS. (1999) 13(11):1373–5. 10.1097/00002030-199907300-00015 10449291

[24] Rodrigues KL Borges JP Lopes GDO Pereira EDS Mediano MFF Farinatti P Influence of physical exercise on advanced glycation End products levels in patients living with the human immunodeficiency virus. Front Physiol. (2018) 9:1641. 10.3389/fphys.2018.01641 30574090 PMC6291474

[25] Vancampfort D Mugisha J Richards J De Hert M Probst M Stubbs B. Physical activity correlates in people living with HIV/AIDS: a systematic review of 45 studies. Disabil Rehabil. (2018) 40(14):1618–29. 10.1080/09638288.2017.1306587 28325087

[26] Rehm KE Konkle-Parker D. Physical activity levels and perceived benefits and barriers to physical activity in HIV-infected women living in the deep south of the United States^†^. AIDS Care. (2016) 28(9):1205–10. 10.1080/09540121.2016.1164802 27023306 PMC4971578

[27] Jaggers JR Hand GA. Health benefits of exercise for people living with HIV: a review of the literature. Am J Lifestyle Med. (2016 May) 10(3):184–92. 10.1177/155982761453875 30202273 PMC6124952

[28] Vancampfort D Mugisha J De Hert M Probst M Firth J Gorczynski P Global physical activity levels among people living with HIV: a systematic review and meta-analysis. Disabil Rehabil. (2018) 40(4):388–97. 10.1080/09638288.2016.1260645 27929355

[29] d’Ettorre G Ceccarelli G Giustini N Mastroianni CM Silvestri G Vullo V. Taming HIV-related inflammation with physical activity: a matter of timing. AIDS Res Hum Retroviruses. (2014) 30(10):936–44. 10.1089/AID.2014.0069 25055246 PMC4179917

[30] Oyeyemi AL Moss SJ Monyeki MA Kruger HS. Measurement of physical activity in urban and rural South African adults: a comparison of two self-report methods. BMC Public Health. (2016) 16(1):1004. 10.1186/s12889-016-3693-6 27658580 PMC5034669

[31] Cook I Alberts M Brits JS Choma SR Mkhonto SS. Descriptive epidemiology of ambulatory activity in rural, black South Africans. Med Sci Sports Exerc. (2010) 42(7):1261–8. 10.1249/MSS.0b013e3181ca787c 20019642

[32] Kruger HS Venter CS Vorster HH Margetts BM. Physical inactivity is the major determinant of obesity in black women in the north west province, South Africa: the THUSA study. Nutrition. (2002) 18(5):422–7. 10.1016/s0899-9007(01)00751-1 11985949

[33] Maimela E Alberts M Modjadji SEP Choma SSR Dikotope SA Ntuli TS The prevalence and determinants of chronic non-communicable disease risk factors amongst adults in the dikgale health demographic and surveillance system (HDSS) site, Limpopo province of South Africa. PLoS One. (2016) 11(2):e0147926. 10.1371/journal.pone.0147926 26882033 PMC4755539

[34] Mabweazara SZ Leach LL Ley C. Physical activity among HIV positive women of low socioeconomic status: benefits and barriers. Afr J Phys Act Health Sci. (2017) 23(4):533–48. ISSN: 2411-6939

[35] Siedner MJ Bibangambah P Kim J Lankowski A Chang JL Yang IT Treated HIV infection and progression of carotid atherosclerosis in rural Uganda: a prospective observational cohort study. J Am Heart Assoc. (2021) 10(12):e019994. 10.1161/JAHA.120.019994 34096320 PMC8477876

[36] Siedner MJ Lankowski A Tsai AC Muzoora C Martin JN Hunt PW GPS-measured distance to clinic, but not self-reported transportation factors, are associated with missed HIV clinic visits in rural Uganda. AIDS. (2013) 27(9):1503–8. 10.1097/QAD.0b013e32835fd873 23435294 PMC3745818

[37] North CM Allen JG Okello S Sentongo R Kakuhikire B Ryan ET HIV infection, pulmonary tuberculosis, and COPD in rural Uganda: a cross-sectional study. Lung. (2018 Feb) 196(1):49–57. 10.1007/s00408-017-0080-8 29260309 PMC6261662

[38] Dziak JJ Dierker LC Abar B. The interpretation of statistical power after the data have been gathered. Curr Psychol. (2020) 39(3):870–7. 10.1007/s12144-018-0018-1 32523323 PMC7286546

[39] Okello S Kim J Sentongo RN Tracy R Tsai AC Kakuhikire B Blood pressure trajectories and the mediated effects of body mass index and HIV-related inflammation in a mixed cohort of people with and without HIV in rural Uganda. J Clin Hypertens. (2019) 21(8):1230–41. 10.1111/jch.13621 PMC6759805 31278845

[40] Muchira J Stuart-Shor E Manne-Goehler J Lo J Tsai AC Kakukire B Validity of hemoglobin A1c for diagnosing diabetes among people with and without HIV in Uganda. Int J STD AIDS. (2019) 30(5):479–85. 10.1177/0956462418823406 30714875 PMC6719298

[41] Filmer D Pritchett LH. Estimating wealth effects without expenditure data-or tears: an application to educational enrollments in states of India. Demography. (2001) 38(1):115. 10.1353/dem.2001.0003 11227840

[42] Bauman A Bull F Chey T Craig CL Ainsworth BE Sallis JF The international prevalence study on physical activity: results from 20 countries. Int J Behav Nutr Phys Act. (2009) 6(1):21. 10.1186/1479-5868-6-21 19335883 PMC2674408

[43] Cleland C Ferguson S Ellis G Hunter RF. Validity of the international physical activity questionnaire (IPAQ) for assessing moderate-to-vigorous physical activity and sedentary behaviour of older adults in the United Kingdom. BMC Med Res Methodol. (2018) 18(1):176. 10.1186/s12874-018-0642-3 30577770 PMC6303992

[44] Craig CL Marshall AL Sjöström M Bauman AE Booth ML Ainsworth BE International physical activity questionnaire: 12-country reliability and validity. Med Sci Sports Exerc. (2003) 35(8):1381–95. 10.1249/01.MSS.0000078924.61453.FB 12900694

[45] Bursac Z Gauss CH Williams DK Hosmer DW. Purposeful selection of variables in logistic regression. Source Code Biol Med. (2008) 3(1):17. 10.1186/1751-0473-3-17 19087314 PMC2633005

[46] Alonso A Barnes AE Guest JL Shah A Shao IY Marconi V. HIV infection and incidence of cardiovascular diseases: an analysis of a large healthcare database. J Am Heart Assoc. (2019) 8(14):e012241. 10.1161/JAHA.119.012241 31266386 PMC6662120

[47] Thivalapill N Simelane T Mthethwa N Dlamini S Lukhele B Okello V Transition to dolutegravir is associated with an increase in the rate of body mass index change in a cohort of virally suppressed adolescents. Clin Infect Dis. (2021) 73(3):e580–6. 10.1093/cid/ciaa1652 33119739 PMC8326552

[48] Chetty L Cobbing S Chetty V. Physical activity and exercise for older people living with HIV: a scoping review. HIVAIDS - Res Palliat Care. (2021) 13:1079–90. 10.2147/HIV.S336886 PMC8702781 34984030

[49] Schuelter-Trevisol F Wolff FH Alencastro PR Grigoletti S Ikeda ML Brandao ABM Physical activity: do patients infected with HIV practice? How much? A systematic review. Curr HIV Res. (2012) 10(6):487–97. 10.2174/157016212802429794 22762420

[50] Tegene Y Mengesha S van der Starre C Lako S Toma A Spigt M. Physical activity level and associated factors among adult HIV patients in Ethiopia. BMC Infect Dis. (2022) 22(1):123. 10.1186/s12879-022-07120-z 35120443 PMC8817526

[51] Chisati EM Constantinou D Lampiao F. Effects of maximal strength training on bone mineral density in people living with HIV and receiving anti-retroviral therapy: a pilot study. BMC Sports Sci Med Rehabil. (2020) 12(1):67. 10.1186/s13102-020-00216-6 33110607 PMC7585307

[52] Kitilya B PrayGod G Peck R Changalucha J Jeremiah K Kavishe BB Levels and correlates of physical activity and capacity among HIV-infected compared to HIV-uninfected individuals. PLoS One. (2022) 17(1):e0262298. 10.1371/journal.pone.0262298 35061774 PMC8782412

[53] Frantz JM Murenzi A. The physical activity levels among people living with human immunodeficiency virus/acquired immunodeficiency syndrome receiving high active antiretroviral therapy in Rwanda. SAHARA-J J Soc Asp HIVAIDS. (2013) 10(3–4):113–8. 10.1080/17290376.2014.886081 PMC4039135 24521093

[54] Hyle EP Martey EB Bekker LG Xu A Parker RA Walensky RP Diet, physical activity, and obesity among ART-experienced people with HIV in South Africa. AIDS Care. (2023) 35(1):71–7. 10.1080/09540121.2021.2012556 34913762 PMC9200895

[55] Roos R Myezwa H Van Aswegen H. “If you have a problem with your heart, you have a problem with your life”: self-perception and behaviour in relation to the risk of ischaemic heart disease in people living with HIV. Afr J Prim Health Care Fam Med. (2015) 7(1):772. 10.4102/phcfm.v7i1.772 26245593 PMC4564874

[56] Eckard AR McComsey GA. Weight gain and integrase inhibitors. Curr Opin Infect Dis. (2020) 33(1):10–9. 10.1097/QCO.0000000000000616 31789693 PMC7433018

[57] Guwatudde D Kirunda BE Wesonga R Mutungi G Kajjura R Kasule H Physical activity levels among adults in Uganda: findings from a countrywide cross-sectional survey. J Phys Act Health. (2016) 13(9):938–45. 10.1123/jpah.2015-0631 27172614

[58] Wachira LJ Hayker SO Larouche R Oyeyemi AL Prista A Owino GE Physical activity and active transportation behaviour among rural, peri-urban and urban children in Kenya, Mozambique and Nigeria: the PAAT study. PLoS One. (2022) 17(1):e0262768. 10.1371/journal.pone.0262768 35061821 PMC8782337

[59] Alemu T Lindtjørn B. Physical activity, illness and nutritional status among adults in a rural Ethiopian community. Int J Epidemiol. (1995) 24(5):977–83. 10.1093/ije/24.5.977 8557456

[60] Shehu RA Abdullahi AA Adekeye DS. Sedentary lifestyle and wellness in Kaduna state, Nigeria. Stud Ethno-Med. (2010) 4(1):15–9. 10.1080/09735070.2010.11886358

[61] Assah FK Ekelund U Brage S Mbanya JC Wareham NJ. Urbanization, physical activity, and metabolic health in sub-Saharan Africa. Diabetes Care. (2011) 34(2):491–6. 10.2337/dc10-0990 21270205 PMC3024374

[62] Mashili FL Kagaruki GB Mbatia J Nanai A Saguti G Maongezi S Physical activity and associated socioeconomic determinants in rural and urban Tanzania: results from the 2012 WHO-STEPS survey. Int J Popul Res. (2018) 2018:1–10. 10.1155/2018/4965193

[63] Mabweazara SZ Leach LL Ley C Onagbiye SO Dave JA Levitt NS Erratum: Demographic and socio-economic predictors of physical activity among people living with HIV of low socio-economic status. Health SA Gesondheid. (2021) 26:1560. Available at: http://www.hsag.co.za/index.php/hsag/article/view/1560. eCollection 2021. 34394966 10.4102/hsag.v26i0.1560 PMC8335763

[64] Malambo P Kengne AP Lambert EV De Villiers A Puoane T. Prevalence and socio-demographic correlates of physical activity levels among South African adults in Cape town and mount frere communities in 2008–2009. Arch Public Health. (2016) 74(1):54. 10.1186/s13690-016-0167-3 28042473 PMC5198503

[65] Mlangeni L Makola L Naidoo I Chibi B Sokhela Z Silimfe Z Factors associated with physical activity in South Africa: evidence from a national population based survey. Open Public Health J. (2018 Dec 20) 11(1):516–25. 10.2174/1874944501811010516

[66] Dang AK Nguyen LH Nguyen AQ Tran BX Tran TT Latkin CA Physical activity among HIV-positive patients receiving antiretroviral therapy in Hanoi and Nam Dinh, Vietnam: a cross-sectional study. BMJ Open. (2018) 8(5):e020688. 10.1136/bmjopen-2017-020688 29748343 PMC5950700

[67] Raka B Gulati, K O'Sullivan MB Rao AS Vinez LM. Levelling the field: improving opportunities for women farmers in Africa. World Bank. Available at: https://documents.worldbank.org/en/publication/documents-reports/documentdetail/579161468007198488/Levelling-the-field-improving-opportunities-for-women-farmers-in-Africa (cited September 16, 2022).

[68] Modola S. workload through labour-saving technologies and practices. 8.

[69] Mengesha MM Roba HS Ayele BH Beyene AS. Level of physical activity among urban adults and the socio-demographic correlates: a population-based cross-sectional study using the global physical activity questionnaire. BMC Public Health. (2019) 19(1):1160. 10.1186/s12889-019-7465-y 31438909 PMC6704679

[70] Oguoma VM Nwose EU Skinner TC Richards RS Digban KA Onyia IC. Association of physical activity with metabolic syndrome in a predominantly rural Nigerian population. Diabetes Metab Syndr Clin Res Rev. (2016) 10(1):13–8. 10.1016/j.dsx.2015.08.010 26327395

[71] Haregu TN Khayeka-Wandabwa C Ngomi N Oti S Egondi T Kyobutungi C. Analysis of patterns of physical activity and sedentary behavior in an urban slum setting in Nairobi, Kenya. J Phys Act Health. (2016) 13(8):830–7. 10.1123/jpah.2015-0510 26998581

[72] Barr AL Partap U Young EH Agoudavi K Balde N Kagaruki GB Sociodemographic inequities associated with participation in leisure-time physical activity in sub-Saharan Africa: an individual participant data meta-analysis. BMC Public Health. (2020) 20(1):927. 10.1186/s12889-020-08987-w 32539702 PMC7296740

[73] Meltzer DO Jena AB. The economics of intense exercise. J Health Econ. (2010) 29(3):347–52. 10.1016/j.jhealeco.2010.03.005 20371127 PMC2864796

[74] Humphreys BR Ruseski JE. Economic determinants of participation in physical activity and sport. Working Papers. International association of sports economists & north American association of sports economists (2006). (Working Papers). Report No.: 0613. Available at: https://ideas.repec.org/p/spe/wpaper/0613.html (cited September 17, 2022).

[75] Shuval K Li Q Gabriel KP Tchernis R. Income, physical activity, sedentary behavior, and the ‘weekend warrior’ among U.S. Adults. Prev Med. (2017) 103:91–7. 10.1016/j.ypmed.2017.07.033 28802654

[76] Puciato D Rozpara M Mynarski W Oleśniewicz P Markiewicz-Patkowska J Dębska M. Physical activity of working-age people in view of their income status. BioMed Res Int. (2018) 2018:1–7. 10.1155/2018/8298527 PMC6236765 30515414

[77] Stalsberg R Pedersen A. Are differences in physical activity across socioeconomic groups associated with choice of physical activity variables to report? Int J Environ Res Public Health. (2018) 15(5):922. 10.3390/ijerph15050922 29734745 PMC5981961

[78] Geleijnse JM. Impact of dietary and lifestyle factors on the prevalence of hypertension in western populations. Eur J Public Health. (2004) 14(3):235–9. 10.1093/eurpub/14.3.235 15369026

[79] Diaz KM Shimbo D. Physical activity and the prevention of hypertension. Curr Hypertens Rep. (2013) 15(6):659–68. 10.1007/s11906-013-0386-8 24052212 PMC3901083

[80] Pescatello LS Franklin BA Fagard R Farquhar WB Kelley GA Ray CA. Exercise and hypertension. Med Sci Sports Exerc. (2004) 36(3):533–53. 10.1249/01.mss.0000115224.88514.3a 15076798

[81] Fagard RH. Exercise therapy in hypertensive cardiovascular disease. Prog Cardiovasc Dis. (2011) 53(6):404–11. 10.1016/j.pcad.2011.03.006 21545926

[82] Cornelissen VA Smart NA. Exercise training for blood pressure: a systematic review and meta-analysis. J Am Heart Assoc. (2013) 2(1):e004473. 10.1161/JAHA.112.004473 23525435 PMC3603230

[83] Carlson DJ Dieberg G Hess NC Millar PJ Smart NA. Isometric exercise training for blood pressure management: a systematic review and meta-analysis. Mayo Clin Proc. (2014) 89(3):327–34. 10.1016/j.mayocp.2013.10.030 24582191

