# Supplementary material for: Correlates of physical activity among people living with and without HIV in rural Uganda
Source: Front Reprod Health. 2023 Jul 20;5:1093298. doi: 10.3389/frph.2023.1093298 (PMC10398393; doi:10.3389/frph.2023.1093298)
Supplement: Supplemental Table 1 — Multivariable linear regression models to identify sociodemographic and clinical correlates of physical activity in people with and without HIV restricted to individuals living in rural areas [file Table1.pdf]

Supplementary Table 1. Multivariable linear regression models to identify sociodemographic and clinical correlates of physical activity in people with and without HIV restricted to individuals living in rural areas

	**Multivariable Model**		
	**β**	**95% C I**	**p-value**
**Age (each year)**	-37	-99, 25	0.238
**Sex**			
Male	REF		
Female	1099	164, 2034	0.021
**Education^**			
Primary or less	REF		
Secondary and greater	-734	-2166, 698	0.314
**Wealth Quartile++**			
Fewest assets	REF		
Few assets	-451	-1675, 772	0.468
Middle assets	-1014	-2255, 227	0.109
Most assets	-3215	-4684, -1746	<0.001
***Diastolic BP(each 10 mmHg)**	-517	-950, -84	0.019
**HIV serostatus**			
HIV uninfected	REF		
PWH	-1634	-2623, -645	0.001

BP= Blood Pressure; **^**A total of 13 (12 HIV-uninfected and 1 HIV-infected) were missing education assessment; + One participant had missing HbA1c assessment; ++We used the mean asset index for each person over the course of the study period and a total of 13 participants were missing wealth quartile data; *Average of second and third same sitting left and right arm blood pressure measurements.
