# Supplementary material for: Correlates of physical activity among people living with and without HIV in rural Uganda
Source: Front Reprod Health. 2023 Jul 20;5:1093298. doi: 10.3389/frph.2023.1093298 (PMC10398393; doi:10.3389/frph.2023.1093298)
Supplement: Supplemental Table 2 — Multivariable linear regression models to identify sociodemographic and clinical correlates of physical activity in people with and without HIV living with interaction terms on sex, BMI, age, mean diastolic blood pressure and serostatus [file Table2.pdf]

Supplementary Table 2. Multivariable linear regression models to identify sociodemographic and clinical correlates of physical activity in people with and without HIV living with interaction terms on sex, BMI, age, mean diastolic blood pressure and serostatus

	Multivariable Model		
	**β**	**95% C I**	**p-value**
**Interaction by Sex**			
Female sex	1834	608.78, 3058.90	0.003
Serostatus HIV+	-950	-2208.56, 308.45	0.138
Sex*Serostatus	-1331	-3049.56, 388.52	0.129
**Interaction by BMI**			
BMI 18.5-25kg/m^2	416	-1235.66, 2067.19	0.621
BMI ≥ 25kg/m^2	2271	236.21, 4306.73	0.029
Serostatus HIV+	-1128	-3726.77, 1471.10	0.394
BMI18.5-25kg/m^2*Serostatus	-221	-2976.01, 2533.91	0.875
BMI ≥ 25kg/m^2*Serostatus	-1556	-4593.00, 1481.87	0.314
**Interaction by Age[#]/year**			
Age > 50	753	-471.59,1977.72	0.227
Serostatus HIV+	-1197	-2561.70,167.49	0.085
Age*Serostatus	-637	-2374.71,1100.66	0.471
**Interaction by DBP(each 10 mmHg)**			
DBP	-46	-97.86,5.75	0.081
Serostatus HIV+	-1464	-7032.91, 4105.65	0.605
DBP *Serostatus	-1	-77.26, 74.49	0.971

All models adjusted for educational attainment, wealth quartile and residential location. DBP=Diastolic Blood Pressure; ^A total of 13 (12 HIV-uninfected and 1 HIV-infected) were missing education assessment; [+] One participant had missing HbA1c assessment; [++]We used the mean asset index for each person over the course of the study period and a total of 13 participants were missing wealth quartile data; *Average of second and third same sitting left and right arm blood pressure measurements. [#]Age tertiles are used with age categorised as 0 = 50 years and below and 1 = >50 years and above.
